# Evaluating the Accuracy of Web-Based and In-Clinic Subjective Cognitive Decline Assessments in Detecting Cognitive Impairment: Multicohort Study

**DOI:** 10.2196/69689

**Published:** 2025-08-11

**Authors:** Jae Myeong Kang, Manchumad Manjavong, Adam Diaz, Miriam T Ashford, Anna Aaronson, Joseph Eichenbaum, Scott Mackin, Rachana Tank, Melanie J Miller, Bernard Landavazo, Erika Cavallone, Diana Truran, Monica R Camacho, Juliet Fockler, Derek Flenniken, Sarah Tomaszewski Farias, Michael W Weiner, Rachel Nosheny

**Affiliations:** 1 VA Advanced Imaging Research Center, San Francisco VA Medical Center, San Francisco, CA, United States; 2 Department of Psychiatry, Gachon University College of Medicine, Gil Medical Center, Incheon, Republic of Korea; 3 Department of Psychiatry and Behavioral Sciences, University of California San Francisco, 4150 Clement Street (114M), San Francisco, CA, 94121, United States; 4 Division of Geriatric Medicine, Department of Internal Medicine, Faculty of Medicine, Khon Kaen University, Khon Kaen, Thailand; 5 Department of Radiology and Biomedical Imaging, University of California San Francisco, San Francisco, CA, United States; 6 Northern California Institute for Research and Education, 4150 Clement Street (151NC), San Francisco, CA, 94121, United States, 1 6504680619, 1 4156682864; 7 Dementia Research Centre, UCL Institute of Neurology, University College London, London, United Kingdom; 8 Department of Neurology, University of California Davis, Sacramento, CA, United States; 9 Department of Medicine, University of California San Francisco, San Francisco, CA, United States

**Keywords:** subjective cognitive decline, ECog-12, online, MCI, dementia, web-based, Everyday Cognition scale, mild cognitive impairment

## Abstract

**Background:**

Scalable tools to efficiently identify individuals likely to have cognitive impairment (CI) are critical in the Alzheimer disease and related dementias field. The Everyday Cognition scale (ECog) and its short form (ECog12) assess subjective cognitive and functional changes and are useful in predicting CI. Recent advances in online technology have enabled the use of web-based cognitive tests and questionnaires to identify CI with greater convenience and scalability. While the effectiveness of the ECog has been demonstrated in clinical settings, its potential to detect CI in remote, unsupervised formats remains underexplored.

**Objective:**

This study aimed to compare the ability of the web-based ECog and the in-clinic ECog in distinguishing between individuals with CI and those who are cognitively unimpaired (CU), and to evaluate the effectiveness of the ECog12—the short version of the ECog—compared to the full-length ECog in a web-based setting.

**Methods:**

Participants were recruited from the Brain Health Registry (BHR; web-based) and Alzheimer’s Disease Neuroimaging Initiative (ADNI; in-clinic) settings with available clinical diagnoses. The ability of the self-reported ECog (Self-ECog), study partner–reported ECog (SP-ECog), Self-ECog12, and SP-ECog12 to discriminate individuals with CI from CU was assessed using receiver operating characteristic (ROC) curves. Area under the ROC curves (AUCs) between BHR and ADNI were compared using the DeLong test, as were AUCs between ECog12 and ECog in BHR.

**Results:**

Web-based Self-ECog and SP-ECog scores effectively discriminated CI from CU with AUCs of 0.722 and 0.818, respectively. Similarly, the abbreviated web-based versions, Self-ECog12 and SP-ECog12, also demonstrated discriminative ability (AUC=0.709 and 0.777, respectively). When compared to in-clinic ECog scores, there were no significant differences in the ability to distinguish CI from CU between web-based and in-clinic versions (BHR Self-ECog AUC=0.722 vs ADNI Self-ECog AUC=0.769, DeLong *P*=.06; BHR SP-ECog AUC=0.818 vs ADNI SP-ECog AUC=0.840, DeLong *P*=.50). Additionally, the comparison between web-based ECog and ECog12 showed no significant difference in AUCs (BHR Self-ECog AUC=0.722 vs BHR Self-ECog12 AUC=0.709, DeLong *P*=.18).

**Conclusions:**

Web-based ECog scores, both the full-length and short-form, were as valid as in-clinic ECog scores for identifying clinically diagnosed CI. In addition, Self-ECog12 was as effective as full-length Self-ECog to identify CI in a web-based setting, offering a cost-effective and accessible screening tool for large-scale studies. These results highlight the value of the web-based ECog as a valid tool for identifying older adults with CI in a remote clinical study, facilitating early detection and referral for comprehensive evaluations for identifying potential candidates for disease-modifying therapy.

## Introduction

Dementia is a syndrome characterized by cognitive and noncognitive symptoms, affecting activities of daily living. Alzheimer disease (AD) is the most common cause of dementia, marked by amyloid beta and tau neuropathology, which leads to memory decline and other cognitive symptoms that precede dementia [1]. The recent development of disease-modifying medications such as aducanumab, lecanemab, and donanemab has brought new therapeutic options into clinical practice [2]. Consequently, identifying individuals likely to have mild cognitive impairment (MCI)—potential candidates for disease-modifying therapy—has become increasingly important.

Neuropsychological tests are widely used as diagnostic tools for cognitive impairment (CI), differentiating between individuals who are cognitively unimpaired (CU), those with MCI, and those with dementia [3]. However, these tests are time-consuming, require trained professionals, and may not effectively capture fluctuations or progressive changes in cognitive function [4]. The Everyday Cognition scale (ECog), developed by Farias et al [5], offers a practical, time-efficient alternative that can be self-administered, focusing on realistic assessments of daily functioning. A key feature of the ECog is its ability to assess subjective cognitive changes compared to 10 years ago, accounting for individual differences in baseline cognitive function. Additionally, it can address anosognosia—a common issue in individuals with MCI—through informant-administered ECog, which correlates more closely with clinical diagnoses than self-administered ECog [5-7]. Past studies have demonstrated the utility of ECog in identifying people with CI or AD pathology and predicting future conversion to MCI or dementia in healthy older adults [5-10].

Despite its proven effectiveness in clinical settings, the potential of ECog in remote, unsupervised formats remains underexplored. The Alzheimer’s Disease Neuroimaging Initiative (ADNI) [11] recently began remote, unsupervised administration of the ECog12, a validated short form of the ECog [7,8], to participants and their study partners (SP) [12]. This shift to digital platforms marks a significant advancement in clinical research, allowing for broader reach and more continuous data collection [12]. Similarly, the Brain Health Registry (BHR), which has remotely administered ECog to over 64,680 participants since 2014 [13], exemplifies the potential of web-based platforms to facilitate large-scale cognitive assessments and long-term monitoring. These platforms [13-15] present significant advantages for identifying individuals at risk of cognitive decline, including the cost- and time-effectiveness of reducing in-person visits. Our study specifically investigates the validity and utility of the web-based ECog as compared to their traditional, in-clinic counterparts.

The overall goal of this study was to determine the ability of the web-based ECog to distinguish individuals with CI from CU. We investigated three hypotheses: (1) the web-based ECog will be associated with clinically diagnosed CI, (2) the web-based ECog will show comparable association with CI as the in-clinic ECog, and (3) the web-based ECog12 will be as effective in identifying CI as the full-length online ECog. These hypotheses were tested by estimating associations between ECog and clinical diagnosis and comparing the accuracy of in-clinic versus web-based ECog and ECog12 versus the full ECog to distinguish diagnostic groups.

## Methods

### Participants

Participants in this study were recruited from two sources: (1) the BHR Electronic Validation of Online Methods to Predict and Monitor Cognitive Decline (eVAL) study [16,17] for the web-based component and (2) the ADNI study for the in-clinic component.

#### BHR eVAL

The BHR is a web-based neuroscience registry dedicated to the evaluation and monitoring of various cognitive disorders in a remote setting. Participants and their invited study partners visit the BHR website to complete remote cognitive assessments every 6 months [7]. The eVAL study is a multisite study that used the BHR platform to validate electronic versions of existing in-clinic cognitive monitoring instruments through the internet-based BHR platform. All eVAL participants were also evaluated in an in-clinic setting [18]. Participants were enrolled in eVAL from BHR at the University of California San Francisco and from the Alzheimer’s Disease Research Centers at the University of Alabama at Birmingham, Mayo Clinic in Rochester, MN, and Washington University [18].

#### ADNI

Data used in the preparation of this study were obtained from the ADNI database [19]. Launched in 2003 as a collaboration between public and private sectors under the leadership of the principal investigator (MWW), ADNI aims to determine if serial magnetic resonance imaging, positron emission tomography scans, and other biological markers, along with clinical and neuropsychological evaluations, can effectively track the progression of MCI and early AD. For the latest updates, visit the ADNI database website [19].

#### Inclusion Criteria

This study included participants from BHR eVAL and ADNI with the following criteria: (1) age between 55 and 90 years; (2) have completed the Self-ECog or SP-ECog questionnaire, or both; (3) have a clinical diagnosis of CU or MCI; and (4) underwent both ECog questionnaire and clinical diagnostic evaluation at the baseline evaluation period.

### Ethical Considerations

This study was approved by the institutional review board of the University of California San Francisco. Participants provided web-based informed consent upon enrollment in the BHR and written informed consent at their first in-clinic visit for enrollment in the eVAL study. For the ADNI study, all study protocols were approved by the institutional review board of the respective participating study centers, and participants provided written informed consent. All data were anonymized. Compensation for participation varied depending on the level and duration of involvement, ranging from US $0 to US $3125 over a 5-year period [20].

### Measurement

#### Everyday Cognition Scale

This study used baseline ECog scores from all participants. ECog is a 39-item questionnaire that assesses subjective changes in cognition and instrumental activities of daily living compared to 10 years ago [5]. Each item is rated on a 1‐4 Likert scale (1, no change or better; 2, questionable or occasionally worse; 3, consistently a little worse; 4, consistently much worse), with the total score being the average of all responses and higher scores indicating greater decline. ECog ratings can be provided either by the individual themselves (self-reported ECog or Self-ECog) or by a study partner (SP-ECog). In this study, SP-ECog was used when available. ECog was assessed in a web-based unsupervised setting in the BHR eVAL study and in an in-clinic setting in the ADNI study.

ECog12 is a short form of ECog that was developed using selected 12 items throughout all cognitive domains [8]. ECog12 has shown a reduced testing time with high consistency and clinical validity, and several previous studies have used ECog12 [6,8,21]. For this study, the full ECog was administered in both BHR and ADNI. We derived the ECog12 score from the full ECog by selecting the 12 items from the original, full-length instrument and calculating the average of the 12 items.

#### Clinical Diagnosis

All participants in this study underwent clinical diagnostic evaluations. In the BHR eVAL study, participants were clinically diagnosed as CU, having MCI, or very mild dementia according to the Uniform Data Set version 3. This standard was developed by the National Alzheimer’s Coordinating Center under the supervision of the National Institute on Aging and is implemented across all U.S. Alzheimer’s Disease Centers [22].

In ADNI, participants were evaluated for an initial diagnosis using scores from the Clinical Dementia Rating (CDR) scale, the Mini Mental State Exam, as well as clinical judgment. Participants were categorized into baseline diagnostic categories as follows: cognitively normal across all ADNI phases (1/GO/2/3), subjective memory concern (SMC) in ADNI 2, early MCI and late MCI in ADNI GO/2, MCI in ADNI 1/3, and AD across all ADNI phases. For this study, cognitively normal, SMC, and MCI (early MCI, late MCI, and MCI) participants were included.

Clinical diagnoses in this study were categorized into two groups: (1) CU, which includes CU from the BHR eVAL study and cognitively normal and SMC from ADNI; and (2) CI, which includes MCI and very mild dementia from the BHR eVAL study and early MCI, late MCI, and MCI from the ADNI study.

#### Demographic and Clinical Information

Participants’ demographic and clinical information were included in this study: age at baseline, gender (male and female), years of education, and race (African American, Asian, White, and other [American Indian/Alaskan Native, Hawaiian/Other Pacific Islander, more than one, and unknown/declined to state race]).

Baseline CDR was obtained from both cohorts [23]. We used the CDR sum of boxes score, which is a reliable and valid diagnostic and dementia staging measure [24], with higher scores indicating a greater level of CI. The Geriatric Depression Scale (GDS) Short Form was also obtained for both studies [25]. One GDS item regarding memory symptoms (“Do you feel you have more problems with memory than most?”) was excluded from the GDS score, thus making the total score ranging from 0 to 14. A higher score indicates more depressive symptoms. Self-reported memory concern was also obtained from both cohorts and was a yes-or-no question: “Are you concerned that you have a memory problem?” in BHR eVAL and “Are you concerned that you have a memory or other thinking problem?” in ADNI.

### Statistical Analysis

#### Descriptive Analysis

We produced descriptive statistics according to the diagnostic groups (CU and CI) and web-based or in-clinic study setting (BHR and ADNI) and compared demographic and clinical information and ECog scores between groups using independent *t* test (2-tailed) and chi-square test.

#### Ability of Web-Based ECog for Predicting CI

To test the first hypothesis that the web-based ECog can distinguish diagnostic groups, we performed binomial logistic regression analyses with the diagnostic group (CU or CI) as the dependent variable. We then calculated the area under the curve (AUC) using the receiver operating characteristic curve. Subsequent metrics including optimal ECog cut point score, sensitivity, specificity, and Youden index were also calculated. Additionally, adjusted AUCs based on the predicted value of a logistic model with ECog score, age, gender, years of education, race, and GDS were calculated.

#### Comparison Between Web-Based and In-Clinic ECog

To compare the ability of web-based ECog for discriminating CI from CU to that of in-clinic ECog, we compared the AUC for discriminating CI from CU in BHR to the AUC in ADNI using the DeLong test.

#### Comparison Between ECog12 and ECog in Web-Based Setting

To compare the effectiveness of web-based ECog12 in distinguishing CI from CU to the full-length web-based ECog, we also compared the AUC of ECog12 and the AUC of full-length ECog in BHR. Statistical significance was set at *P*<.05, there was no correction for multiple comparisons, and R software (version 4.3.2; R Foundation for Statistical Computing) was used in all analyses.

## Results

### Participants

#### Demographic and Clinical Information

A total of 1801 participants were included in this study (n=428 in BHR; n=1373 in ADNI). Table 1 presents the demographic and clinical information of participants, including comparisons across diagnostic groups and between the BHR and ADNI cohorts. Differences between CU and CI were observed for gender (*χ*^2^_1_=6, *P*=.01), CDR sum of boxes (*t*_77.9_=–7.37, *P*<.001), and self-reported memory concern (*χ*^2^_1_=37.6, *P*<.001) in BHR. In ADNI, differences were found in all demographic and clinical variables except for age. BHR participants showed higher GDS scores in both CU (*t*_394.5_=14.51, *P*<.001) and CI (*t*_70.0_=5.80, *P*<.001) groups compared to ADNI.

**Table 1.** Participant characteristics.

	BHR^a^				ADNI^b^				BHR vs ADNI			
	CU^c^ (n=344)	CI^d^ (n=84)	*t* test (*df*)^e^ or *χ*^2^ (*df*)^f^	*P* value	CU (n=666)	CI (n=707)	*t* test (*df*) or *χ*^2^ (*df*)	*P* value	CU		CI	
									*t* test (*df*) or *χ*^2^ (*df*)	*P* value	*t* test (*df*) or *χ*^2^ (*df*)	*P* value
Age, mean (SD)	71.47 (7.36)	72.75 (7.20)	–1.46 (128.7)^e^	.15	70.99 (6.70)	71.65 (7.50)	–1.72 (1362.7)^e^	.09	1.00 (639.7)^e^	.32	1.32 (105.7)^e^	.19
Gender (female), n (%)	193 (56.1)	34 (40.5)	6.0 (1)^f^	.01	398 (59.8)	317 (44.8)	30.0 (1)^f^	<.001	–1.1 (1)^f^	.29	–0.4 (1)^f^	.52
Education years, mean (SD)	16.94 (2.06)	16.67 (2.47)	0.91 (108.0)^e^	.36	16.68 (2.37)	16.17 (2.58)	3.80 (1369.8)^e^	<.001	1.78 (763.3)^e^	.08	1.69 (101.0)^e^	.09
Race, n (%)			18.4 (3)^f^	.07			30.1 (3)^f^	<.001	–7.97 (3)^f^	.05	–8.9 (3)^f^	.03
African American	23 (6.7)	3 (3.6)			82 (12.3)	42 (5.9)						
Asian	15 (4.4)	2 (2.4)			26 (3.9)	10 (1.4)						
White	293 (85.2)	71 (84.5)			538 (80.8)	632 (89.4)						
Others	13 (3.7)	8 (9.5)			20 (3.0)	23 (3.3)						
CDR-SOB^g^, mean (SD)	0.07 (0.28)	1.08 (1.24)	–7.37 (77.9)^e^	<.001	0.04 (0.14)	1.46 (0.95)	–39.20 (739.8)^e^	<.001	1.76 (436.0)^e^	.08	–2.75 (96.1)^e^	.007
GDS^h^, mean (SD)	2.33 (1.89)	2.77 (2.11)	–1.56 (88.8)^e^	.12	0.06 (1.04)	1.24 (1.34)	–8.93 (1322.3)^e^	<.001	14.51 (394.5)^e^	<.001	5.80 (70.0)^e^	<.001
Self-reported memory concern, n (%)	103 (29.9)	51 (60.7)	37.6 (1)^f^	<.001	269 (40.4)	495 (70.0)	293.6 (1)^f^	<.001	–3.3 (1)^f^	.07	–7.1 (1)^f^	.008
ECog^i^, mean (SD)												
Self-ECog^j^	1.36 (0.39)	1.67 (0.52)	–4.56 (81.1)^e^	<.001	1.37 (0.33)	1.82 (0.57)	–18.13 (1150.3)^e^	<.001	–0.28 (518.7)^e^	.78	–2.21 (80.1)^e^	.03
SP-ECog^k^	1.16 (0.25)	1.72 (0.64)	–7.12 (73.3)^e^	<.001	1.17 (0.25)	1.74 (0.61)	–22.69 (925.8)^e^	<.001	–0.83 (505.9)^e^	.41	–0.29 (80.7)^e^	.77
Self-ECog12^l^	1.34 (0.39)	1.64 (0.54)	–4.27 (80.3)^e^	<.001	1.35 (0.34)	1.77 (0.58)	–16.52 (1150.9)^e^	<.001	–0.40 (517.7)^e^	.69	–1.80 (79.2)^e^	.08
SP-ECog12	1.17 (0.27)	1.69 (0.66)	–6.38 (73.9)^e^	<.001	1.18 (0.27)	1.73 (0.62)	–21.34 (957.9)^e^	<.001	–0.55 (507.2)^e^	.58	–0.47 (80.2)^e^	.64

a BHR: Brain Health Registry.

b ADNI: Alzheimer’s Disease Neuroimaging Initiative.

c CU: cognitively unimpaired.

d CI: cognitively impaired.

e *t* test (*df*)

f *χ*2 (*df*)

g CDR-SOB: Clinical Dementia Rating – Sum of Boxes.

h GDS: Geriatric Depression Scale.

i ECog: Everyday Cognition scale.

j Self-ECog: self-reported ECog.

k SP-ECog: study partner–reported ECog.

l ECog12: short version of ECog.

#### ECog Scores in Both Groups

Table 1 displays ECog scores. In all cases, Self-ECog scores tended to be higher than SP-ECog scores, although not statistically proven. The only significant difference between ADNI and BHR was observed in Self-ECog scores within the CI group (ADNI > BHR; *t*_80.1_=–2.21, *P*=.03).

### Association Between ECog Scores and CI

Table 2 presents the AUC values for distinguishing CI from CU in BHR and ADNI. The AUCs were moderate in BHR (0.709‐0.818) and ADNI (0.747‐0.840). After adjusting for age, gender, years of education, race, and GDS, the AUCs remained fair to moderate in both BHR and ADNI (0.724‐0.851). Subsequent metrics including optimal cut point, sensitivity, specificity, and diagnostic accuracy are also presented in Table 2 for both studies. In adjusted AUC, a linear regression model was used with predictors including ECog score, age, gender, years of education, race, and GDS.

**Table 2.** ECog^a^ AUCs^b^ for discriminating CI^c^ from CU^d^.

	AUC (confidence interval)	Adjusted AUC	Cut point	Sensitivity	Specificity	Accuracy	Youden index
In BHR^e^							
Self-ECog^f^	0.722 (0.660‐0.783)	0.736	≥1.361	0.742	0.663	0.677	0.406
SP-ECog^g^	0.818 (0.759‐0.878)	0.807	≥1.263	0.725	0.791	0.777	0.515
Self-ECog12^h^	0.709 (0.646‐0.772)	0.724	≥1.364	0.652	0.680	0.675	0.331
SP-ECog12	0.777 (0.759‐0.878)	0.770	≥1.273	0.652	0.780	0.754	0.432
In ADNI^i^							
Self-ECog	0.769 (0.743‐0.792)	0.796	≥1.436	0.731	0.683	0.708	0.414
SP-ECog	0.840 (0.819‐0.860)	0.851	≥1.231	0.792	0.722	0.758	0.514
Self-ECog12	0.747 (0.720‐0.772)	0.782	≥1.375	0.724	0.665	0.696	0.389
SP-ECog12	0.817 (0.794‐0.839)	0.834	≥1.273	0.732	0.758	0.744	0.489

a ECog: Everyday Cognition scale.

b AUC: area under the curve.

c CI: cognitively impaired.

d CU: cognitively unimpaired.

e BHR: Brain Health Registry.

f Self-ECog: self-reported ECog.

g SP-ECog: study partner–reported ECog.

h Self-ECog12: short version of ECog.

i ADNI: Alzheimer’s Disease Neuroimaging Initiative.

### Comparing AUCs Between BHR and ADNI

**Figure 1.**
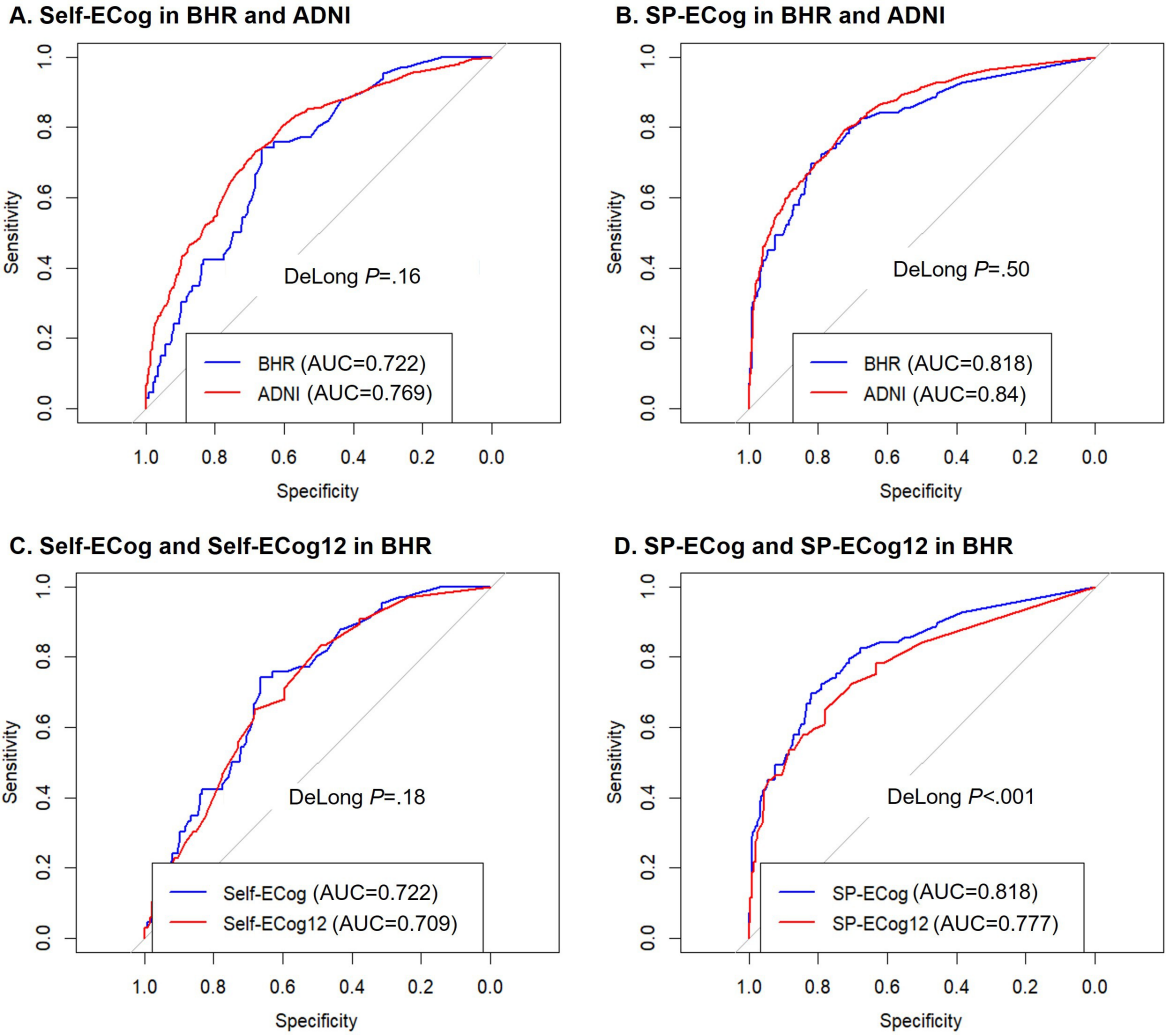
Receiver operating curves for predicting cognitive impairment. ADNI: Alzheimer’s Disease Neuroimaging Initiative; AUC: area under the curve; BHR: Brain Health Registry; ECog: Everyday Cognition scale; ECog12: short version of ECog; Self-ECog: self-reported ECog; SP-ECog: study partner–reported ECog.

Figure 1 illustrates the AUCs of BHR and ADNI and their comparisons. Both Self-ECog (BHR AUC=0.722, confidence interval 0.660‐0.783; ADNI AUC=0.769, confidence interval 0.743‐0.792; DeLong *P*=.16) and SP-ECog (BHR AUC=0.818, confidence interval 0.759‐0.878; ADNI AUC=0.840, confidence interval 0.819‐0.860; DeLong *P*=.5) showed comparable AUC values between BHR and ADNI.

### Comparing AUCs Between ECog and ECog12 in BHR

Figure 1 depicts the comparison between ECog and ECog12 in BHR. Self-ECog and Self-ECog12 demonstrated comparable AUCs (Self-ECog AUC=0.722, Self-ECog12 AUC=0.709, DeLong *P*=.18), while SP-ECog exhibited a higher AUC than SP-ECog12 (SP-ECog AUC=0.818, SP-ECog12 AUC=0.777, DeLong *P*<.001).

## Discussion

### Principal Results

This study investigated the effectiveness of a measure of subjective cognitive decline, ECog, to detect CI in two cohorts: web-based in BHR and in-clinic in ADNI. The first finding was that the ECog demonstrated moderate accuracy in distinguishing individuals with CI and CU in both BHR and ADNI. The second finding was that the web-based ECog in BHR showed a comparable ability to detect CI, similar to the in-clinic ECog scores in ADNI. The third finding was that the ECog12, the short form of the ECog, was as effective as the full-length ECog for detecting CI in the BHR. These results indicate that ECog scores, whether administered web-based or in-clinic, and in both short-form and full-length versions, are useful in identifying individuals clinically diagnosed with CI.

The first finding of this study is that the web-based ECog demonstrated a moderate association with CI. Both online Self- and SP-ECog scores effectively distinguished individuals clinically diagnosed with MCI and CU. This result aligns with previous research that has shown a strong association between web-based SP-ECog and self-reported diagnoses of MCI or AD [7]. Unlike prior research that relied solely on either remotely collected data [7,26] or in-clinic data [6,8,9], our analysis included individuals with both web-based ECog data and clinical evaluations, allowing for a more rigorous assessment of the relationship between web-based subjective cognitive decline and objectively diagnosed CI. This finding highlights the potential clinical utility of online subjective cognitive decline measures, suggesting that they may reflect underlying cognitive impairment as determined by expert clinical evaluation. Previous in-clinic studies have reported that informant-reported ECog scores are better at predicting CI from CU than self-reported scores [6,8,9]. A prior study focusing on older adults with CI found that web-based SP-ECog could distinguish AD dementia from MCI, while Self-ECog could not [27]. The ability of both Self- and SP-ECog to identify CI in this study might be due to the fact that most participants in BHR had mild cognitive symptoms [28], which makes it less likely that they are unaware of their cognitive status [29]. It is also noteworthy that establishing web-based ECog cut point scores with good sensitivities, specificities, and accuracies is useful as a screening tool to detect CI in the older adult population. Our findings demonstrate the utility of web-based ECog for detecting the clinical diagnosis of CI, particularly in mildly impaired populations.

The second finding is that web-based ECog and in-clinic ECog produced comparable results in discriminating CI from CU. Previous studies have found that online ECog scores closely correspond with in-clinic ECog scores [30]. Our study newly revealed that web-based ECog is as effective as in-clinic ECog for detecting CI from CU, as demonstrated by the AUC comparison, which showed no significant difference between the two measures. This finding suggests that remotely collected ECog can serve as a viable alternative to in-clinic ECog for cognitive screening, expanding accessibility to cognitive assessments beyond traditional clinical settings. The trend toward overall higher discriminative AUC values, although not significant, in ADNI compared to BHR might be attributed to the nature of web-based scales, including response quality, lower engagement due to convenience, and potential technical issues [31-33]. From a clinical perspective, however, the lack of statistical significance in the AUC comparison suggests that both web-based and in-clinic ECog can be considered equally effective for CI screening, with the web-based version offering the added benefits of convenience and accessibility, particularly in populations with limited access to in-person assessments.

The third finding is that Self-ECog12 performed comparably to full-length Self-ECog in distinguishing CI from CU in a remote setting. ECog12, derived from the original 39-item ECog, maintains good psychometric properties for various cognitive domains, including memory, visuospatial function, language, and executive function [8]. Previous studies have shown that ECog12 is as effective as full-length ECog in identifying the risk of MCI [6,9]. The fact that web-based ECog12 shows a similar association with the diagnosis of CI versus CU as the 39-item ECog supports its use in web-based clinical studies like BHR. Recent large-scale neuroscience studies have started collecting online data from questionnaires about cognitive function, leveraging extensive and longitudinal collections [34-36]. ADNI 4, the current phase of ADNI, also recently implemented web-based ECog12 in a novel online screening approach to aid in selecting participants for plasma AD biomarker analysis and in-clinic evaluation [12,21]. However, SP-ECog12 showed a significantly lower probability of detecting CI from CU compared to SP-ECog in this study, although both SP-ECog and SP-ECog12 showed moderate AUCs. We believe this discrepancy may be due to the brevity and the reduced amount of information collected by study partners in the web-based ECog12. This might be further supported by the fact that study partners, who may not know the participants well or see them frequently, can still take part in the BHR study [7]. Nevertheless, the web-based ECog12 remains a useful and time-efficient tool for identifying individuals at risk of MCI in remote settings.

Recently, advances in online technology have allowed the use of web-based cognitive tests to identify CI with convenience and scalability [37,38]. Self-administered web-based digital neuropsychological tests are an effective method for identifying CI, offering benefits such as accessibility, efficiency, and diagnostic accuracy for dementia [39]. Many well-known in-clinic tests, including the Montreal Cognitive Assessment, Cogstate Brief Battery, and Cambridge Neuropsychological Test Automated Battery (CANTAB, Cambridge Cognition Ltd), have been adapted for online use [40-43]. Although neuropsychological tests objectively measure cognitive abilities through rigorous standardized tasks, ECog, which is a brief measure of subjective cognitive decline, captures an individual’s or their informant’s perception of cognitive and functional changes over the past 10 years in real life. This can provide early indicators of cognitive decline that objective tests might miss [5,29]. As ECog and ECog12 can be easily administered in a remote setting with validity for identifying CI from CU, integrating both objective and subjective measures of cognitive decline in web-based studies could enhance early detection of MCI who are potential candidates for disease-modifying therapy and comprehensive understanding of cognitive health.

### Limitations

This study has several limitations. First, the BHR eVAL study and ADNI consist predominantly of participants with over 16 years of education, 85% of whom are non-Latinx White. This lack of demographic diversity likely limits the generalizability of the findings and introduces bias related to differences in race, ethnicity, cognitive reserve, and socioeconomic status—factors that can influence the diagnosis, prognosis, and treatment response in dementia. African American and Latinx older adults are up to 1.5 to 2 times more likely to develop AD and AD-related dementias compared to White older adults [44]. These disparities may stem from differences in medical risk factors for AD (eg, depression, diabetes, obesity, and hypertension), biological factors (eg, genetics, epigenetics, or proteomic markers), factors contributing to cognitive reserve (eg, education and occupation), and nonbiological or social factors (eg, economic stability, safety, housing, and environment) [12]. Additionally, BHR has limitations, including selection bias, low data integrity, informant variability, and high dropout rates. Participants are more likely to have higher digital proficiency and increased awareness of cognitive health issues, further skewing the representativeness of the sample. On the other hand, web-based studies offer the advantage of scalability, enabling participation from individuals who might otherwise be unable to join due to geographic or time constraints. However, challenges such as data inaccuracy, missing information, and the inability to verify whether participants completed assessments independently or with assistance remain. Another important limitation is the difficulty in managing participant dropout over time. Prior studies have also shown that non-White or Latinx individuals, as well as those with lower educational attainment, are more likely to discontinue participation in follow-up assessments in web-based settings [45]. These patterns highlight the need for more inclusive recruitment strategies and efforts to retain diverse populations in future studies to ensure findings are more broadly applicable.

The second limitation is that this study was not originally designed to compare web-based and in-clinic populations. Instead, it compared these populations retrospectively, meaning that the recruitment process was not perfectly controlled from the outset, and there may be participation self-selection biases. Third, the cross-sectional design of this study limits the interpretation of the results due to the lack of longitudinal cause-and-effect relationships between ECog scores and clinical diagnoses. Fourth, the lack of correction for multiple comparisons is an additional limitation of the study.

### Conclusions

This study highlights the value of web-based ECog as an effective and scalable tool for identifying older adults at risk of MCI, particularly in web-based settings. Subjective cognitive changes measured in a remote, unsupervised, online setting distinguished CI from CU diagnostic groups with moderate accuracy. Compared to in-clinic scales, the web-based ECog showed a comparable ability to discriminate CI from CU. ECog12, a shorter form developed to increase brevity, also demonstrated a moderate association with clinical diagnosis in a remote setting. These results highlight the value of the web-based ECog as a valid tool for identifying older adults with MCI in an online clinical study, facilitating early detection and referral for more comprehensive evaluations in both research and clinical settings.

## Abbreviations

AD: Alzheimer disease
ADNI: Alzheimer’s Disease Neuroimaging Initiative
AUC: area under the curve
BHR: Brain Health Registry
CDR: Clinical Dementia Rating
CI: cognitively impaired
CU: cognitively unimpaired
ECog: Everyday Cognition Scale
ECog12: short version of ECog
eVAL: Electronic Validation of Online Methods to Predict and Monitor Cognitive Decline
GDS: Geriatric Depression Scale
MCI: mild cognitive impairment
ROC: receiver operating characteristic
Self-ECog: self-reported ECog
SMC: subjective memory concern
SP-ECog: study partner–reported ECog
